# Ruptured Hepatocellular Carcinoma: A Case Report

**DOI:** 10.7759/cureus.64365

**Published:** 2024-07-11

**Authors:** Sonal Prasad, Jay Xiong, Loui Abdelghani

**Affiliations:** 1 Department of Internal Medicine, St. Joseph's Medical Center, Stockton, USA; 2 Department of Pulmonary and Critical Care Medicine, St. Joseph's Medical Center, Stockton, USA

**Keywords:** spontaneous hemoperitoneum, transarterial embolization (tae), transarterial radioembolization, liver rupture, hepatocellular carcinoma (hcc)

## Abstract

Hepatocellular carcinoma (HCC) is one of the most common primary liver tumors in the world. In the United States, it is very uncommon for the liver mass to spontaneously rupture, especially if it has already been treated with embolization. Prompt diagnosis and treatment are necessary to improve the overall prognosis. Unfortunately, even with treatment, the patient can still rapidly decline. We present a case of a patient who was diagnosed with HCC and received treatment with transarterial radioembolization (TARE) with yttrium-90 (Y90). Despite this, the patient’s liver mass grew and spontaneously ruptured. Although the patient received additional embolizations for his mass, he still deteriorated and eventually expired.

## Introduction

Hepatocellular carcinoma (HCC) is one of the most common cancers in the world. It makes up more than 90% of primary liver cancer. It typically occurs in about 85% of cirrhotic patients. Risk factors for HCC are viral hepatitis, alcoholic liver disease, and non-alcoholic fatty liver disease [1]. The etiology of HCC also plays an important role in its prognosis. For example, the hepatitis B virus has the most positive impact on overall survival [2]. Serum alpha-fetoprotein is a biomarker that is often elevated in advanced HCC. Hepatocellular carcinoma may be diagnosed with ultrasound, computed tomography (CT), or magnetic resonance imaging (MRI); liver biopsy is not routinely performed due to the risk of tumor seeding and bleeding [1]. Spontaneous rupture of HCC is uncommon, with an incidence of less than 3% in Western countries. Its incidence is much higher in Asia and Africa, where it can range from 3% to 26% [3]. Even patients who have already received treatment for their HCC with either transarterial embolization (TAE) or hepatic resection, can still present with a liver mass rupture. For example, in a study of 294 patients who received embolization, two patients presented with ruptured HCC following treatment, with an incidence of 0.68% [4]. In another study with 351 patients, three patients presented with tumor rupture with an incidence of 0.85% [5]. In this article, we report an uncommon case of a patient in the United States with a history of HCC status post-TARE with yttrium-90 (Y90) who presented with a ruptured HCC and unfortunately expired despite aggressive management.

## Case presentation

**Figure 1:**
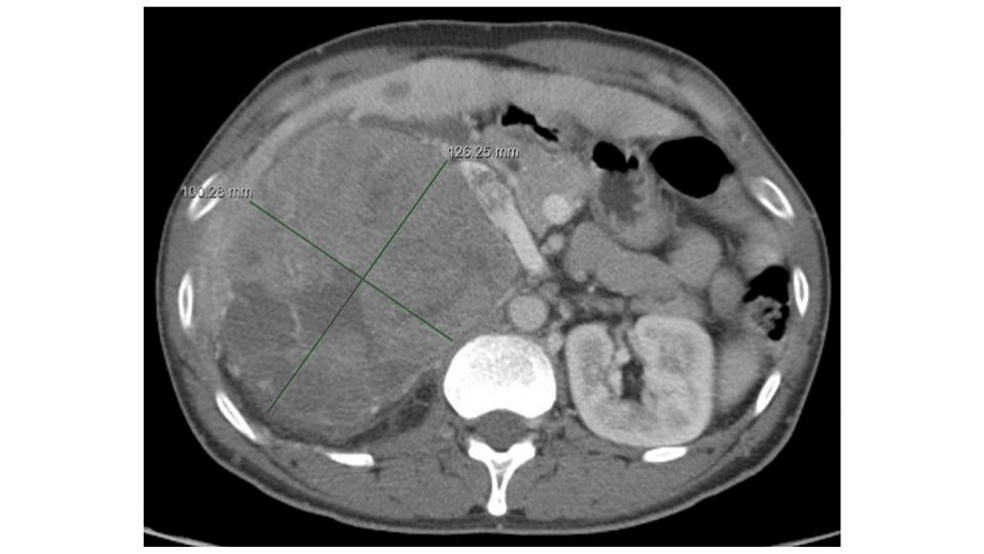
Axial view of the CT scan demonstrates a large right abdominal heterogeneous enhancing mass measuring 10.0 cm x 12.6 cm in length and width.

**Figure 2:**
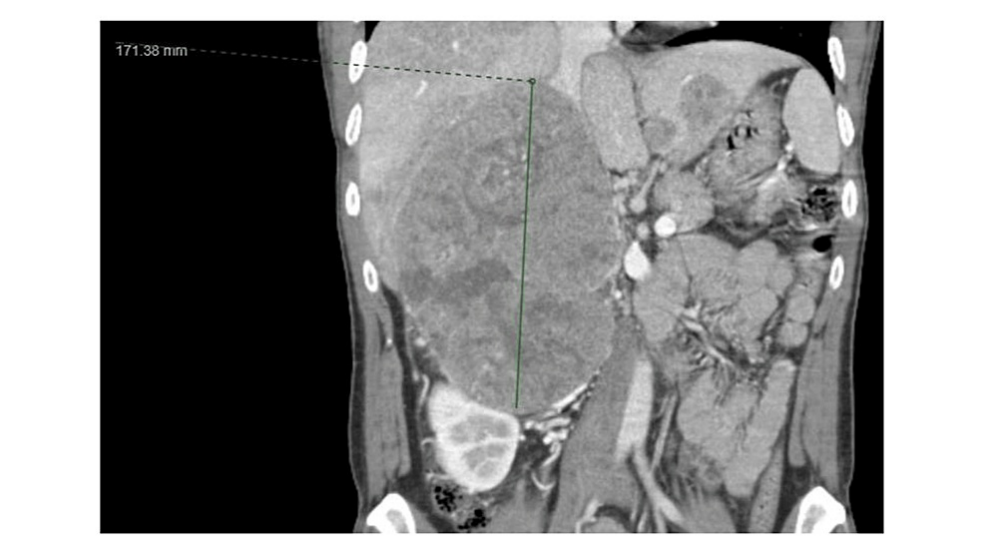
Coronal view of the CT scan demonstrates a large right abdominal heterogeneous enhancing mass measuring 17.1 cm in height.

**Figure 3:**
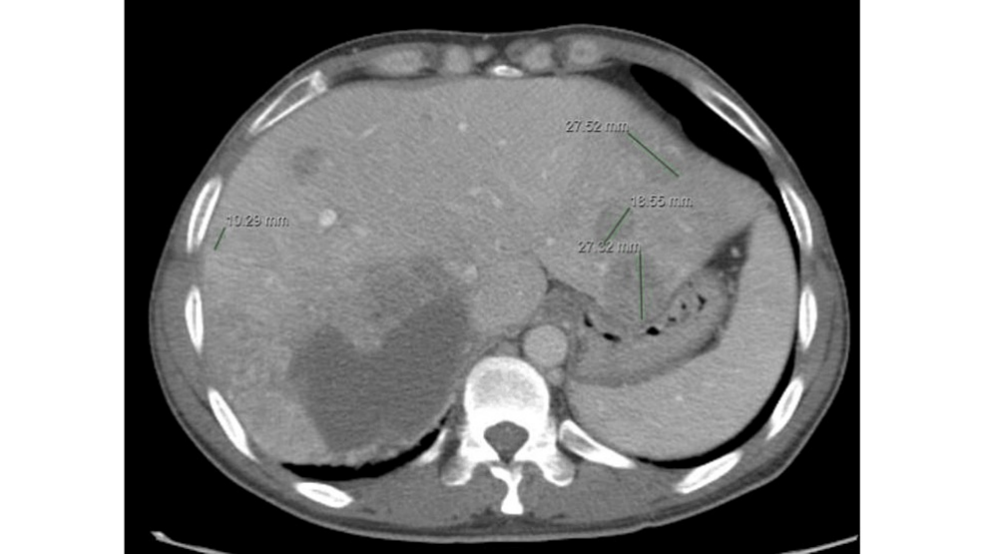
Axial view of the CT scan demonstrates multiple hepatic lesions in the left and right hepatic lobes.

A 46-year-old male with a history of hepatitis B and HCC presented with severe abdominal pain. Nine months prior, the patient was diagnosed with biopsy-proven HCC and subsequently underwent TARE with Y90 about four months later. He then relocated to the West Coast from the South and established care with a local oncologist. The patient complained of chronic right upper quadrant pain that was associated with nausea, vomiting, fatigue, and loss of appetite. His oncologist ordered a CT scan of the chest, abdomen, and pelvis using intravenous (IV) contrast. The CT scan revealed a large right abdominal heterogeneous enhancing mass, measuring 10.0 cm x 12.6 cm x 17.1 cm (Figures 1, 2), along with multiple hepatic lesions in the left and right hepatic lobes (Figure 3). The patient was referred to an interventional radiologist (IR) for liver-directed therapy.

During his visit with his IR, the patient's case was classified as Barcelona Clinic Liver Cancer stage B, Eastern Cooperative Oncology Group Performance Status grade 1, and Child-Pugh class A. He was not a surgical candidate due to his underlying multifocal liver masses. Options for liver-directed therapy include percutaneous transarterial chemoembolization (TACE) or TARE. Chemoembolization was deemed to be a poor choice for this patient, as he would likely not be able to tolerate it. Therefore, it was recommended that he undergo palliative Y90 radioembolization treatment. 

Unfortunately, before the patient could make it to his appointment for the liver-directed therapy, he was admitted to the hospital for severe abdominal pain. Initial vitals showed a temperature of 36.5 °C, a heart rate of 107 beats per minute, a respiratory rate of 24 breaths per minute, saturation of 100% on room air, and a blood pressure of 118/64 mmHg. His complete blood count, coagulation panel, and lactic acid were as follows: white blood cell count of 11.0 thousand/uL, hemoglobin of 10.0 g/dL, hematocrit of 32.3%, platelet count of 391 thousand/uL, lactic acid of 3.4 mmol/L, international normalized ratio (INR) of 1.3, prothrombin time (PT) of 14.6 sec, and partial thromboplastin time (PTT) of 26.3 sec.

**Figure 4:**
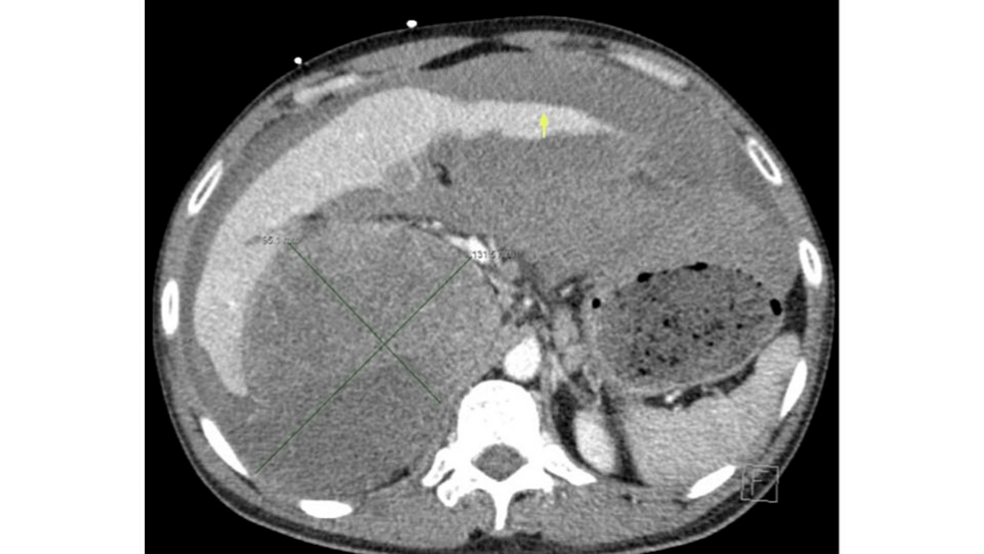
Axial view of the CT demonstrates a large right abdominal heterogeneous enhancing mass measuring 9.5 cm x 13.1 cm in length and width. The yellow arrow points to the hemoperitoneum.

**Figure 5:**
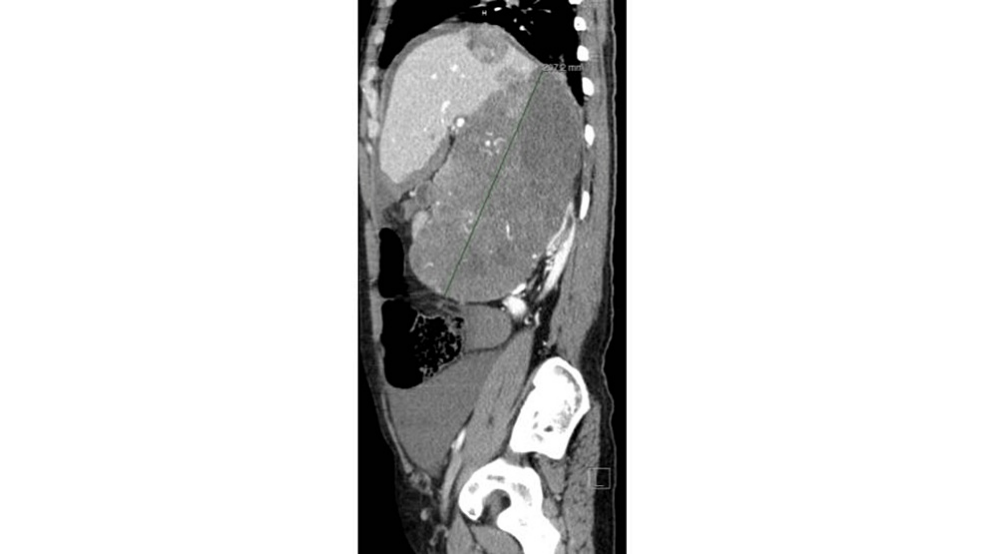
Sagittal view of the CT demonstrates a large right abdominal heterogeneous enhancing mass measuring 20.7 cm in height.

The patient received a CT scan of the abdomen and pelvis with IV contrast to evaluate his right upper abdominal pain. It revealed the same large heterogeneously enhancing mass that is similar in size compared to the images from about a month prior (Figures 1, 2), measuring approximately 9.5 cm x 13.1 cm x 20.7 cm (Figures 4, 5). However, there was now a concern for the interval development of a large complex of partially hyperattenuating ascites, with suspicion for hemoperitoneum (Figure 4). It was possible that this was related to a ruptured and actively bleeding metastasis at the left edge of the left lobe of the liver laterally, with a possible focus on active contrast extravasation. Therefore, IR, general surgery, and intensivist were immediately consulted.

Repeat hemoglobin and hematocrit were ordered. Hemoglobin dropped from 10.0 g/dL to 8.0 g/dL within four hours. The patient also became tachycardic in the 150s and tachypneic in the 30s, but his blood pressure was still hemodynamically stable. The IR immediately took the patient for a hepatic angiogram and embolization. The HCC was ruptured, but there was no active contrast extravasation identified. Empiric gel foam and particle embolization of the multifocal HCC were performed. Afterward, he received two units of packed red blood cells (pRBC) and two units of fresh frozen plasma (FFP). Despite this intervention, the patient progressively deteriorated. He slowly became oliguric, with a repeat basic metabolic panel revealing worsening renal function suspected to be secondary to tubular injury in the setting of contrast-mediated nephropathy. He also became hemodynamically unstable, requiring four vasopressors. He needed dialysis but was not a candidate due to the significant shock and underlying malignancy. His hemoglobin would also continuously drop and then be replaced with adult blood transfusions. Unfortunately, he was so unstable that he could not get a repeat CT of his abdomen and pelvis to re-evaluate his intra-abdominal bleeding. His oxygen saturation worsened to the point where he needed to be intubated. On day four, he had a cardiac arrest with the initial rhythm of asystole. The return of spontaneous circulation was achieved in seven minutes. Then his wife changed his code status to “do not resuscitate.” Two hours later, he had another episode of asystole and expired.

## Discussion

Hepatocellular carcinoma is a common cancer worldwide that has an incidence rate of less than 3% in Western countries and spontaneously ruptures and leads to life-threatening complications [3]. Hepatic rupture is the third cause of death in HCC after tumor progression and liver failure. Risk factors for spontaneous HCC rupture are cirrhosis, hypertension, extra-hepatic invasion, ascites, and liver mass that is greater than 5 cm in size [6]. Our patient only had one of these criteria, and that was a liver mass measuring approximately 9.5 cm x 13.1 cm x 20.7 cm. 

If HCC is diagnosed early, the prognosis is as high as 70% for a five-year survival rate, but if it is diagnosed during an advanced stage, the survival rate will be less than 20%. The prognosis is also affected by the etiology of the tumor. For example, in a 2020 study, it was shown that the etiology of the hepatitis B virus showed a higher median survival time, followed by the hepatitis C virus, metabolic disorders, and finally alcoholic liver disease [2]. Our patient had a history of hepatitis B, and therefore, it was highly likely that this was the etiology of the HCC. 

There is no single guideline for the management of patients with a ruptured HCC. Treatment is generally personalized to the specific individual. Management consists of conservative treatment, TAE/TACE, hepatic resection, or a combination of TAE/TACE and hepatic resection [6]. Conservative management has been shown to be poor, with a mortality rate of 85%-100% and a median survival of about 13 days [7]. In hemodynamically unstable patients, the mainstay treatment is to stabilize the patient and correct the hemorrhagic shock with hemostasis. TAE/TACE is less invasive than hepatic resection, with a success rate of 53% to 100%. It also has a lower 30-day mortality rate [3]. Emergent hepatic resection is usually not recommended in unstable patients because of its high periprocedural mortality. Elective hepatic resection has more favorable results [6]. Unfortunately, in the case of our patient, he was not a surgical candidate for elective hepatic resection due to his underlying multifocal liver masses. Despite a history of TARE with Y90 five months ago and another embolization during this admission, our patient continued to worsen. He became so unstable that we were not able to get any further imaging of his abdomen. Additionally, general surgery refused to operate on him and deferred him to a goals-of-care discussion with his family for comfort care.

## Conclusions

Although ruptured HCC is uncommon in Western countries, it can still occur regardless of whether the patient is treated, such as in the case of our patient, who was status post-TARE five months ago. This highlights the need for continued surveillance of the mass despite treatment. Our patient’s liver mass grew within a month and eventually ruptured. Also, in spite of receiving additional embolization during the new hospital admission, he continued to deteriorate with multi-organ failure. Although there are no current guidelines for the management of ruptured HCC, our case emphasizes that the best management is to prevent the rupture from happening because, once ruptured, the prognosis is poor regardless of conservative management versus TAE/TACE versus hepatic resection versus a combination of TAE/TACE and hepatic resection.
